# The Influence of Metabolic Risk Factors on the Inflammatory Response Triggered by Myocardial Infarction: Bridging Pathophysiology to Treatment

**DOI:** 10.3390/cells13131125

**Published:** 2024-06-29

**Authors:** Lisaidy Ramos-Regalado, Sebastià Alcover, Lina Badimon, Gemma Vilahur

**Affiliations:** 1 Research Institute, Hospital de la Santa Creu i Sant Pau, IIB-Sant Pau, 08025 Barcelona, Spainsalcover@santpau.cat (S.A.);; 2 Faculty of Biology, Universitat de Barcelona, 08028 Barcelona, Spain; 3 Ciber CV, Institute Carlos III, 28029 Madrid, Spain; 4 Cardiovascular Research Chair, Universitat Autònoma de Barcelona (UAB), 08193 Barcelona, Spain

**Keywords:** myocardial infarction, inflammatory response, innate immune response, bone marrow, spleen, metabolic risk factors, lifestyle modifications, pharmacological interventions

## Abstract

Myocardial infarction (MI) sets off a complex inflammatory cascade that is crucial for effective cardiac healing and scar formation. Yet, if this response becomes excessive or uncontrolled, it can lead to cardiovascular complications. This review aims to provide a comprehensive overview of the tightly regulated local inflammatory response triggered in the early post-MI phase involving cardiomyocytes, (myo)fibroblasts, endothelial cells, and infiltrating immune cells. Next, we explore how the bone marrow and extramedullary hematopoiesis (such as in the spleen) contribute to sustaining immune cell supply at a cardiac level. Lastly, we discuss recent findings on how metabolic cardiovascular risk factors, including hypercholesterolemia, hypertriglyceridemia, diabetes, and hypertension, disrupt this immunological response and explore the potential modulatory effects of lifestyle habits and pharmacological interventions. Understanding how different metabolic risk factors influence the inflammatory response triggered by MI and unraveling the underlying molecular and cellular mechanisms may pave the way for developing personalized therapeutic approaches based on the patient’s metabolic profile. Similarly, delving deeper into the impact of lifestyle modifications on the inflammatory response post-MI is crucial. These insights may enable the adoption of more effective strategies to manage post-MI inflammation and improve cardiovascular health outcomes in a holistic manner.

## 1. Introduction

Atherothrombosis-induced myocardial infarction (MI) is the leading cause of death worldwide [1,2]. MI triggers a complex and tightly regulated inflammatory response that plays a critical role in the healing process following the ischemic insult [3,4,5]. An exaggerated or deregulated inflammatory response after MI leads to adverse cardiac remodeling and arrhythmias [6].

In recent years, the prevalence of metabolic cardiovascular risk factors, including hypertension, impaired glucose regulation, and dyslipidemia, has increased globally [7]. These factors account for more than 90% of the risk of MI [8] and also modulate the healing process post-event [9,10].

This review aims to provide a comprehensive overview of the complex and tightly regulated local and systemic inflammatory responses triggered by MI and the current knowledge on the deleterious impact of metabolic risk factors in the overall immune process.

## 2. Local Inflammatory Response Triggered by Myocardial Infarction

**Figure 1:**
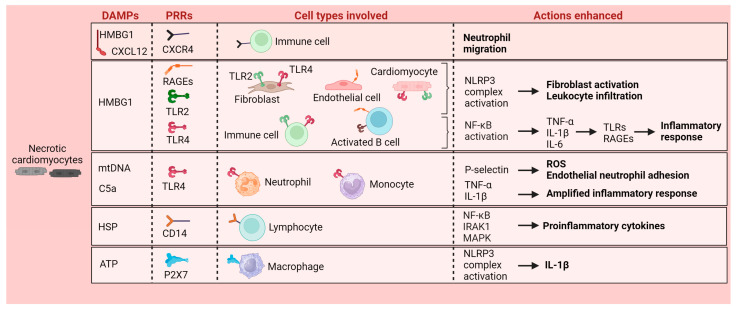
Necrotic cardiomyocytes generate danger-associated molecular patterns (DAMPs) acting as danger signals on different immune cells through pattern recognition receptors (PRRs), enhancing cytokine release and local inflammatory response. High-mobility group box 1 protein (HMGB1); toll-like receptors (TLRs); receptors for advanced glycation end products (RAGEs); heat-shock proteins (HSPs); tumor necrosis factor-alpha (TNF-α); interleukin (IL); mitochondrial DNA (mtDNA); nuclear factor-kappa light chain enhancer of activated B cells (NFκB); receptor-associated kinase 1 (IRAK1); mitogen-activated protein kinase (MAPK); Nucleotide-binding Oligomerization Domain (NOD)-like receptors pyrin domain containing 3 (NLRP3). Illustration created with BioRender.

MI induces the sudden death of myocardial tissue and triggers an intense and regulated inflammatory reaction aimed at repairing the damaged heart [3,11]. Danger-associated molecular patterns (DAMPs) are crucial in this acute inflammatory phase of MI. DAMPs act as danger signals by binding to pattern recognition receptors (PRRs) on immune and stromal cells to initiate the innate immune response [12] (Figure 1). One of the most studied DAMPs is the high-mobility group box 1 protein (HMGB1), a non-histone DNA-binding protein [13]. At the onset of MI, HMGB1 is released by necrotic cells after membrane disruption; once in the extracellular space, HMGB1 binds to chemokine motif ligand 12 (CXCL12), which, in turn, interacts more strongly with CXC receptor type 4 (CXCR4), thus promoting the massive migration of neutrophils to the site of injury [13]. HMGB1 is also a strong ligand for toll-like receptors (TLRs) and receptors for advanced glycation end products (RAGEs), both PRRs. In the ischemic heart, cell surface TLR2 and TLR4 on cardiomyocytes, fibroblasts, and immune cells and RAGEs on inflammatory and endothelial cells (ECs) are the main triggers of the MI-mediated inflammatory response [4,14,15]. Studies in knockout mice subjected to ischemia–reperfusion (I/R) have revealed that deleting TLRs or RAGE receptors reduces the post-MI inflammatory response, decreases infarct size, and lowers oxidative stress [16,17,18,19]. Conversely, HMGB1-related activation of both receptors phosphorylates nuclear factor-kappa light chain enhancer of activated B cells (NFκB), which triggers the transcription of multiple proinflammatory cytokines, including tumor necrosis factor-α (TNF-α), interleukin 1β (IL-1β), and IL-6, and, in turn, upregulates the expression of TLRs and RAGE receptors, further sustaining and amplifying the inflammatory response [13,20]. TLRs and HMGB1 also stimulate the synthesis of CC and CXC chemokines during the acute inflammatory phase post-MI [21]. Furthermore, injured cardiomyocytes release mitochondrial DNA (mtDNA), which is extremely cytotoxic to cardiomyocytes [22] and perpetuates the inflammatory response through TLRs [23]. Serum mtDNA levels have been considered biomarkers of myocardial damage severity following cardiovascular events [24,25]. The release of mtDNA and the overexpression of complement-related mRNA activate the complement system, triggering a cascade of reactions that play a key role in leukocyte recruitment and infiltration in the infarcted heart [26]. mRNA and proteins for all of the components of the classical complement pathway are upregulated in infarcted hearts [11]. One of the complement components, C5a, promotes neutrophil activation and induces neutrophil P-selectin expression, reinforcing endothelial neutrophil adhesion and oxidative stress. C5a also induces monocyte generation of proinflammatory chemokines and cytokines (e.g., TNF-α and IL-1β), overall amplifying the cardiac inflammatory response [3,27].

Nucleotide-binding Oligomerization Domain (NOD)-like receptor (NLR) inflammasomes, particularly NLRP3, also play a critical role in the local immune response. NLR inflammasomes are macromolecular protein complexes that mediate the inflammatory response upon DAMP signaling [14]. Experimental and human studies have supported that the activation of the NLRP3 complex in ECs and cardiomyocytes in the ischemic and border cardiac zones in an early stage of MI leads to fibroblast activation and leukocyte infiltration [28].

Heat-shock proteins (HSPs) and ATP activate the ischemic heart’s immune system. In the extracellular milieu, HSPs bind to TLR4 receptors on the nearest cells and to CD14 of lymphocytes, which leads to the upregulation of NFκB, IL-1 receptor-associated kinase 1 (IRAK1), and p38/mitogen-activated protein kinase (MAPK) pathways, thus increasing the expression of proinflammatory cytokines [29]. On the other hand, in stressed cells, ATP stored in the cytoplasm is massively translocated to the pericellular space and exerts immunomodulatory functions through ligand–ion channel receptors, such as the P2X7 receptor [30]. The ATP/P2X7 axis has been shown, in turn, to trigger the activation of the NLRP3 inflammasome and the subsequent synthesis of IL-1β in monocytes/macrophages subjected to ischemia [31].

Epigenetic changes also contribute to the inflammatory response in the setting of MI. Over the past decade, among microRNAs (miRs), miR-21, miR-33, miR-34a, miR-146a, and miR-155 have been the most extensively studied as “inflamma-miRs” [32]. miR21, miR-33, miR-146a, and miR-155 have been shown to modulate the inflammatory response in MI [33], whereas miR-33 and miR-34a have been more closely associated with fibrosis [32]. Beyond their functions, miRs have been utilized as diagnostic and therapeutic tools, a concept recently termed “theranoMiRNAs” [34,35]. On the other hand, targeting DNA methylation with an inhibitor of DNA methyltransferase (5-azacytidine) has been shown to limit the number of macrophages expressing inducible nitric oxide synthase (iNOS) and, conversely, enhance the number of anti-inflammatory macrophages in the infarcted heart. Additionally, in mice subjected to MI, reduced DNA methylation levels at the *SPI1* promoter CpG island (a proto-oncogene and key player in heart failure post-MI) were observed. This reduction leads to increased gene transcription, which subsequently activates the TLR4/NFκB pathway, resulting in heightened inflammation and cardiomyocyte apoptosis [36]. The role of DNA methylation patterns as potential biomarkers of MI was also investigated in an epigenome-wide association study on acute MI. The study identified 34 novel CpG sites associated with MI. However, their clinical utility as predictive biomarkers or drug targets has not yet been established [36].

## 3. The Involvement of the Bone Marrow and Spleen in the Systemic Inflammatory Response following Myocardial Infarction

In concurrence with the local inflammatory response, MI triggers a systemic inflammatory response that involves bone marrow activation and spleen monocytopoiesis, sustaining the supply of immune cells at the site of cardiac damage [37,38].

**Figure 2:**
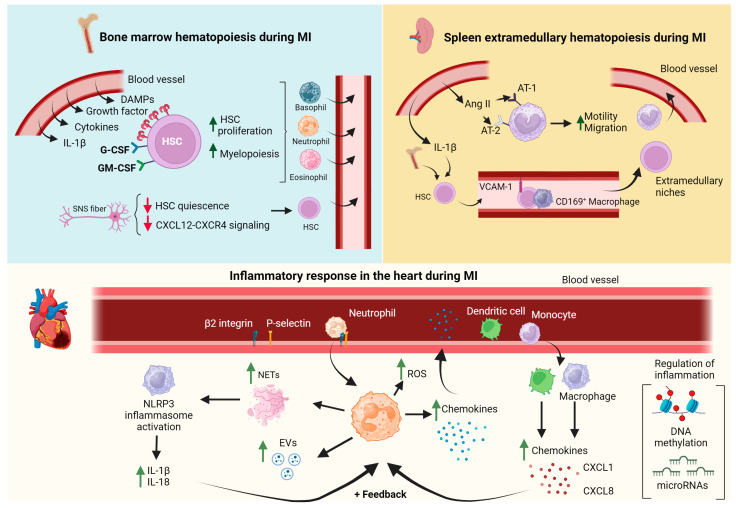
Bone marrow and splenic hematopoiesis fuel the cardiac inflammatory response during myocardial infarction (MI). In the bone marrow, granulocyte colony-stimulating factor (G-CSF) and granulocyte–macrophage colony-stimulating factor (GM-CSF), along with various inflammatory mediators, stimulate the proliferation and release of granulocytes and monocytes into the bloodstream. This process is further supported by sympathetic nervous system fibers since they decrease chemokine motif ligand 12 (CXCL12) activity, promoting Hematopoietic stem cells (HSC) release. In the spleen, angiotensin II (Ang II) enhances the motility and migration of resident monocyte populations, while interleukin-1β (IL-1β) triggers extramedullary hematopoiesis. This process involves the interaction of bone marrow-released HSCs with CD169+ macrophages via vascular cell adhesion molecule 1 (VCAM-1). Consequently, inflammatory cells migrate into the infarcted heart. Neutrophils infiltrate the cardiac tissue through β2-integrin and P-selectin, producing reactive oxygen species (ROS) and chemokines that facilitate the transmigration of dendritic cells and monocytes into the damaged tissue, creating a positive feedback loop. Additionally, neutrophils also release extracellular traps (NETs) and extracellular vesicles (EVs), further sustaining the inflammatory response. Epigenetic modifications, including microRNAs and DNA methylation, also contribute to the modulation of the overall inflammatory response post-MI. Danger-associated molecular patterns (DAMPs); interleukin (IL); angiotensin receptor (AT-1; AT-2); chemokine (C-C motif) ligand 7 (CCL7). Illustration created with BioRender.

The bone marrow is a central organ that produces blood cells through hematopoiesis, a process by which the hematopoietic stem cells (HSCs) asymmetrically divide and maintain the stem cell pool and give rise to different lineage progenitors. The bone marrow niche for HSC mainly comprises mesenchymal stromal cells and ECs [39]. Upon ischemic cardiac injury, HSCs begin their massive production of immune cells, which exit the bone marrow, enter the circulation, and reach the injured heart via adhesion, rolling, and extravasation. The activation of bone marrow hematopoiesis is partially due to DAMPs, growth factors, and cytokines released by the ischemic heart [40] (Figure 2). For instance, IL-1β promotes the proliferation of HSCs through direct effects on hematopoietic cells; granulocyte colony-stimulating factor (G-CSF) induces the maturation, survival, proliferation, activation, and mobilization of granulocytes from the bone marrow into the peripheral circulation [41,42]; and granulocyte–macrophage colony-stimulating factor (GM-CSF) enhances bone marrow myelopoiesis [43]. A recent study has suggested that ADP may also serve as a danger signaling for the hematopoietic bone marrow compartment and foster emergency hematopoiesis after MI through P2Y12-dependent signaling. These data propose a novel, non-canonical role for P2Y_12_ antagonists beyond the inhibition of platelet-mediated atherothrombosis [44]. MI is also associated with the release of vascular endothelial growth factor receptor 2 (VEGFR2), IL-6, and versican from bone marrow ECs, inducing bone marrow endothelial dysfunction and favoring local inflammatory cytokine release [45].

The bone marrow sympathetic nervous system (SNS) also contributes to emergency hematopoiesis [46]. During MI, enhanced SNS activity attenuates HSC quiescence and impairs CXCL12-CXCR4 signaling, thereby altering the proliferation and retention of HSCs [47] and myeloid and lymphoid progenitors [42,48]. The released progenitors may then seed the spleen, yielding a sustained boost in monocyte production [49].

To meet the high leukocyte demand, hematopoiesis can also occur outside the bone marrow, specifically in the spleen (i.e., extramedullary hematopoiesis) [50]. The spleen is a secondary lymphopoietic organ with high metabolic activity and displays several essential functions, such as the maturation of red blood cells, the removal of abnormal cells by phagocytosis, iron recycling, metabolic homeostasis, and humoral and cellular immunity [51]. In addition to B cells, macrophages, and dendritic cells, bona fide undifferentiated monocytes reside in the spleen and outnumber their equivalents in circulation [52]. During the first day post-MI, monocytes residing in the spleen increase their motility and emigrate to the injured heart in a process guided by angiotensin-II signaling [53]. The sustained need for newly made monocytes for the resolution of MI and the recovery of the splenic pool is promoted by extramedullary hematopoiesis, a process partially regulated by IL-1β [54]. Bone marrow-released HSCs, in response to increased sympathetic activity, are retained by CD169+ macrophages via vascular cell adhesion molecule 1 (VCAM-1), leading to the formation of this extramedullary niche [55]. While several studies have demonstrated splenic hematopoiesis in animal models, in humans, this process has so far only been indirectly identified by positron emission tomography imaging of splenic fluor-deoxyglucose uptake (monocytes are highly glycolytic cells) in patients with acute coronary syndromes and in postmortem autopsies of MI patients [56]. Although the systemic increase in innate immune cells has been extensively studied in recent years, the bone marrow and splenic regulation of the adaptive immune response in the setting of MI remains largely unexplored.

## 4. The Migration and Recruitment of Immune Cells to the Infarcted Heart

### 4.1. Neutrophils

The chemokine signaling that culminates in the bone marrow and spleen hematopoietic response after MI stimulates neutrophil mobilization to the heart. Once there, neutrophils interact with endothelial P-selectins, favoring their rolling over the vasculature. Thereafter, neutrophil binding with endothelial β2 integrin triggers a cellular conformational change with the subsequent rearrangement of surface chemokine receptors that enhance and strengthen neutrophil–vasculature interaction. In parallel, junction adhesion molecules are weakened, allowing the infiltration of neutrophils into the underlying tissue, where they induce direct cytotoxic damage to cardiomyocytes by generating reactive oxygen species (ROS) through the activation of myeloperoxidases and the NADP oxidase system [57,58]. Moreover, neutrophils release chemokines that increase the recruitment of other inflammatory cells, such as macrophages and dendritic cells (DCs), to the injury site, which, in turn, release chemokines that favor neutrophil survival (e.g., CXCL1 and CXCL8 suppress neutrophils apoptosis), thus establishing a positive feedback cycle that assures a sustained inflammatory response [59]. Neutrophils also release extracellular traps (NETs) consisting of chromatin filaments fused with granular and cytoplasmatic components, which activate the macrophage NLRP3 inflammasome with the subsequent synthesis of IL-1β and IL-18. IL-1β and IL-18 plasma levels have been shown to positively correlate with the occurrence of adverse cardiac events, infarct size, and cardiac dysfunction in patients with MI [60]. Emerging evidence suggests that neutrophil-derived alarmins (i.e., S100A8/A9) are released during NET formation. S100A8/A9 has been suggested as a dual promoter of inflammation and cardiac repair. Therefore, the effectiveness of S100A8/A9 blockade resides in identifying the optimal therapeutic time window. Neutrophils have also been shown to release extracellular vesicles (EVs). Although the role of neutrophil-derived EVs in the setting of MI has yet to be fully determined [61], EVs from ST-elevation MI (STEMI) patients are found to be enriched with multiple inflammatory mediators.

### 4.2. Monocyte/Macrophages

Early after MI (around 30 min), systemic levels of circulating monocytes increase due to their release from bone marrow and splenic reservoirs [62]. The damaged cardiac ECs produce ROS and monocyte chemoattractant protein-1 (MCP-1 or CCL2), a potent monocyte chemoattractant, favoring monocyte recruitment to the infarcted site [63,64]. MCP-1 and its receptor, C-C motif chemokine receptor 2 (CCR2), are key elements in leukocytosis [65]. In mice, Ly-6C^High^ proinflammatory monocytes from the bone marrow and the spleen are among the first cells to arrive at the infarct zone and reach their peak at day 3 post-MI [66]. From days 1 to 3, monocytes differentiate into mature macrophages that release proinflammatory cytokines (TNF-α, IL-1β) and synthesize adhesion molecules (VCAM1 and P-selectin) [67]. Cardiac macrophages can be distinguished based on the expression of CCR2, recruited CCR2^+^ macrophages, and resident CCR2^−^ (hematopoietic and embryonic origins, respectively) [68]. Activated CCR2^+^ macrophages exert inflammatory effects. As such, they induce monocyte recruitment to the injured heart and promote their differentiation toward macrophages with a proinflammatory phenotype [69]. Comparatively, little is known about the functions of CCR2^−^ macrophages. Studies that deplete mouse CD169^+^ cells (macrophages) prior to I/R injury suggest that resident CCR2^−^ macrophages do not secrete inflammatory mediators but rather activate pathways that inhibit leukocyte recruitment [70].

**Figure 3:**
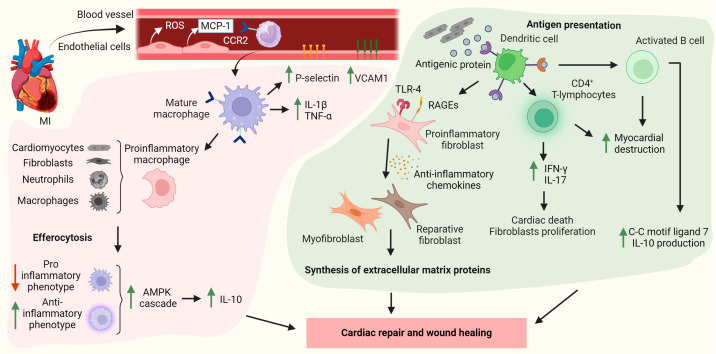
The role of macrophages, dendritic cells, and lymphocytes in the healing process post-myocardial infarction (MI). Monocytes are recruited to the infarcted heart, where they differentiate into activated macrophages, increasing immune cell recruitment and promoting the inflammatory response. Over time, macrophages remove necrotic cells and shift toward an anti-inflammatory phenotype, producing interleukin 10 (IL-10). Dendritic cells present antigenic proteins that shift the fibroblast phenotype toward a proinflammatory state, producing extracellular matrix proteins to maintain myocardial integrity. T and B lymphocyte activation triggers myocardial destruction and interferon gamma (IFN-γ) and IL-17 release. Activated B cells increase C-C motif ligand 7 and release IL-10, which enhance cardiac repair and wound healing. Reactive oxygen species (ROS); monocyte chemoattractant protein-1 (MCP-1); C-C motif chemokine receptor 2 (CCR2); vascular cell adhesion molecule 1 (VCAM-1); tumor necrosis factor-alpha (TNF-α); toll-like receptors (TLRs); receptors for advanced glycation end products (RAGEs). Illustration created with BioRender.

Proinflammatory macrophages are initially responsible for removing necrotic cardiomyocytes and fibroblasts along with apoptotic neutrophils and monocytes post-MI, a process known as efferocytosis [71]. Efferocytosis, in turn, favors the activation of the AMP kinase (AMPK) cascade, promoting a shift in macrophages toward an anti-inflammatory phenotype [72]. The ratio between pro- and anti-inflammatory macrophages in the infarcted region varies throughout the days after the ischemic event. Initially, macrophages secrete proinflammatory cytokines, increasing the inflammatory response and facilitating collagen and extracellular matrix breakdown. Macrophage-related phagocytosis is a crucial step for cardiac wound healing [73]. The regulation of this inflammatory response (time course and magnitude) has a significant impact on cardiomyocyte survival, systolic function, ventricular wall integrity, and fibrosis [4,74]. Once the initial inflammatory response declines, a second wave of monocytosis occurs 7 days post-MI. In mice, Ly-6C^High^ monocytes are recruited to the infarcted areas and undergo differentiation into anti-inflammatory macrophages that secrete IL-10, a cytokine that promotes angiogenesis and cardiac remodeling [75] (Figure 3). Monocyte-derived macrophage populations may undergo further differentiation into various subtypes, some of which are nearly identical to resident cardiac macrophages (CCR2^−^), but a recent investigation suggests that they do not have the same phenotypic specificity that ensures a proper infarct healing process with the formation of a functional scar [76]. Multiple factors regulate macrophage proliferation, recruitment, polarization, and anti-inflammatory activities. A comprehensive bioinformatics analysis has reported ten strongly interlinked hub genes (*Timp1*, *Sparc*, *Spp1*, *Tgfb1*, *Decr1*, *Vim*, *Serpine1*, *Serpina3n*, *Thbs2*, and *Vcan*) that may influence the ventricular remodeling of non-infarcted tissue by modulating fibrosis, macrophage-driven inflammation, and fatty acid metabolism. Specifically, *Vcan* has been suggested to contribute to macrophage activation and promote cytokine release, whereas *Vim* suppresses macrophages’ ROS production, and *Sparc* deletion in infarcted mice leads to a reduction in macrophage content in the remote cardiac region [77]. A recent study in a mouse model of I/R injury has also identified a novel subtype of monocytes named lipid-associated macrophages (SPP1^+^ LAM) within the infarcted area. This discovery has shed light on the role of SPP1^+^ LAM in lipid metabolism and their influence on cardiac remodeling by modulating the MAPK pathway [78]. Whether lipid metabolic alterations may impact SPP1^+^ LAM content deserves to be investigated.

### 4.3. Dendritic Cells

The release of antigenic proteins from necrosed cardiomyocytes induces DC activation. The role of DCs in monocyte recruitment and cardiac repair post-MI has been previously explored in DC-depleted mouse models. DC depletion was associated with a reduction in macrophage content 7 days after MI, followed by reduced infarct size and improved cardiac function [79]. On the other hand, DCs can also directly activate fibroblasts. Cardiac fibroblasts are key players in post-MI cardiac repair and dramatically switch their phenotype during the different phases of myocardial healing [80] (Figure 3). During the inflammatory phase, fibroblasts release proinflammatory cytokines due to TLR, RAGE, and NLRP3 activation [81]. Through the proliferative phase, reparative fibroblasts and differentiated myofibroblasts synthesize extracellular matrix proteins (to prevent ventricle rupture) and are converted into specialized cells to preserve the scar [82]. Cytokines, chemokines, and growth factors released by anti-inflammatory macrophages have a crucial role in the fibroblast phenotype switching to myofibroblast (Figure 3) [67].

### 4.4. Lymphocytes

Thus far, minimal focus has been directed toward T-lymphocytes in the context of MI. Scientific evidence suggests that T cells’ primary protective function is mediated by T-regulatory lymphocytes, which suppress inflammatory processes and activate fibrosis [83]. Moreover, CD4^+^ T-lymphocytes have been suggested to modulate injury size post-MI through the secretion of interferon-γ (IFN-γ) and IL-17, cytokines that stimulate cardiac death and the proliferation of fibroblast [84]. At the same time, however, MI may trigger autoimmune destruction of the myocardial tissue, favoring the activation of autoreactive T-lymphocyte clones [85]. In this regard, CD8^+^ T cells may have a beneficial or detrimental effect at the onset of MI. The deficiency of cytotoxic T cells has been associated with better restoration of heart physiology. Yet, mice lacking cytotoxic T cells died post-MI due to myocardial rupture, which was associated with increased inflammation [86]. B lymphocytes are the most abundant lymphoid cells in human and mouse hearts [87,88], and, like T cells, their functions may be a two-sided coin. As such, B cells produce antibodies that may increase the destruction of the myocardial tissue. Studies blocking IgM have led to a significant reduction in I/R injury [89]. In addition to antibodies, B cells secrete cytokines and chemokines. The production of C-C motif ligand 7, which attracts Ly-6C^High^ monocytes to the myocardium, is dependent on B cells, and the depletion of Ly-6C^High^ monocytes using anti-CD20 antibodies has been shown to interfere with monocyte recruitment, leading to smaller infarcts and a favorable heart remodeling process [90]. One study described a population of B cells with anti-inflammatory properties able to synthesize IL-10, yet the mechanism behind it still remains to be determined (Figure 3) [91].

## 5. The Impact of Metabolic Cardiovascular Risk Factors on the Myocardial Infarction-Induced Inflammatory Response

Hypercholesterolemia, hypertriglyceridemia, type 2 diabetes (T2D), and hypertension are well-known metabolic cardiovascular risk factors associated with the activation of immune cells and may accordingly interfere with the inflammatory healing process post-MI.

The impact of hypercholesterolemia on the MI-induced inflammatory response remains controversial and not fully understood. In APO*3-Leiden mice, hypercholesterolemia was associated with peripheral monocytosis (mostly Ly-6C^High^ monocytes) prior to ischemia, yet lower cardiac macrophage accumulation was detected post-MI. Surprisingly, the infarct size was significantly decreased in hypercholesterolemic mice compared to their normocholesterolemic counterparts [92]. In another study, infarct size was comparable between normo- and hypercholesterolemic rats, yet left ventricle remodeling and the risk of developing heart failure were worse in those animals fed a hypercholesterolemic diet [93]. Similarly, rabbits subjected to I/R and fed a hypercholesterolemic diet exhibited a similar degree of MI damage compared with normal-fed rabbits [94]. In contrast, studies conducted in large preclinical animal models have supported a detrimental effect of hypercholesterolemia on cardiac damage post-MI—leading to an enhanced inflammatory reaction and infarct size [9,95]. Moreover, elevated cholesterol levels have been demonstrated to diminish the effectiveness of several cardioprotective approaches. For instance, ischemic postconditioning, involving brief periods of ischemia and reperfusion following a prolonged ischemic insult [89], notably enhances endothelial function and reduces tissue necrosis in minipigs consuming a standard diet. However, this benefit was not reported in minipigs with hypercholesterolemia [96,97]. Similarly, high cholesterol levels have been found to hinder the anti-inflammatory and antioxidative properties associated with high-density lipoprotein (HDL) particles [98,99], consequently diminishing their cardiovascular protective abilities [98,100,101,102]. Nevertheless, it is noteworthy that returning to normal physiological cholesterol levels through the adoption of a diet low in cholesterol has been demonstrated to reverse HDL dysfunction [103].

Triglyceride-rich lipoproteins (TGRLs) have gained much attention within the last few years. Elevated triglyceride levels have been shown to pose a significant risk for cardiovascular disease and are associated with heightened inflammation [104,105]. Plasma TGs are carried in the blood by chylomicrons and very low-density lipoproteins (VLDLs), collectivity referred to as TGRLs. The breakdown of these TGRLs by lipoprotein lipase (LPL) generates free fatty acids, which can potentially induce cellular damage and stimulate the release of inflammatory mediators [106]. Additionally, macrophages release LPL, which facilitates the hydrolysis of TGRLs, consequently initiating an inflammatory response [107]. A recent observational study found that a combination of elevated remnant cholesterol (the cholesterol content of TGRLs) and low-grade inflammation, indicated by increased C-reactive protein (CRP) levels, conferred the highest risk of MI [108]. However, limited data are available concerning the influence of triglycerides (TGs) on the inflammatory response induced by MI. A study conducted on C57Bl/6 mice fed a high-fructose diet revealed the overexpression of the NLRP3 inflammasome, marked caspase-1 activation, and compromised activation of the cardioprotective reperfusion injury salvage kinase (RIS) and hypoxia-inducible factor (HIF) 2α pathways [109] in the infarcted heart, leading to larger infarcts as compared to mice fed a standard diet. 

Furthermore, TGRLs have been demonstrated to promote endothelial dysfunction, facilitating the adhesion and recruitment of inflammatory cells at the onset of MI [110]. As observed for hypercholesterolemia, the protective effects of ischemic (pre)conditioning were abrogated in fructose-fed hypertriglyceridemic Wistar rats [111].

In diabetes, the precise mechanisms through which disrupted glucose metabolism influences inflammation are not yet fully understood. Prolonged hyperglycemia initiates and advances a non-enzymatic glycation process involving proteins, lipids, and nucleic acids, resulting in the overproduction of ROS. ROS can harm ECs, leading to their activation and dysfunction, subsequently prompting the release of inflammatory mediators [112,113]. Additionally, insulin resistance has been shown to worsen ischemic damage to the myocardium due to a change in cardiac metabolism characterized by a decrease in glucose utilization in favor of free fatty acid oxidation. This shift results in heightened oxygen demand and subsequent contractile dysfunction [114]. The diabetic heart has shown elevated uptake and oxidation of fatty acids, partially influenced by increased activity of peroxisome proliferator-activated receptor alpha (PPARα) triggered by ROS [115]. In turn, PPARα prompts the nuclear translocation of the forkhead box O1 (FOXO1) transcription factor, promoting the progression of cardiomyopathy [116]. Enhanced expression of NLRP3 in monocytes has been observed in patients with T2D [117], while T2D rats have exhibited heightened activation of NLRP3, resulting in intensified cardiac inflammation, cell death, disrupted ultrastructure, and fibrosis [118].

There is a correlation between hypertension and increased mortality following STEMI [119]. Nonetheless, a recent meta-analysis that pooled data from seven randomized clinical trials involving STEMI patients indicated that hypertension did not correlate with larger infarctions or microvascular obstruction, both of which are pathological conditions linked to inflammation [120]. Yet, experimental data have evidenced that the sustained elevation of systemic blood pressure damages the endothelium, disrupting nitric oxide synthesis and inducing ROS production [121]. The weakened blood vessel wall becomes more permeable, favoring the infiltration of inflammatory cells from the bloodstream into the damaged infarcted hearts [122]. Moreover, during the onset of MI, angiotensin II (a significant contributor to hypertension) has been shown to influence the inflammatory process by binding to angiotensin receptors on monocytes and macrophages. This binding stimulates their migration to the site of infarction and subsequent activation, leading to the release of proinflammatory cytokines (Figure 2) [123]. Similarly, Bandoni and colleagues showed that cholinergic stimulation in spontaneously hypertensive rats (SHRs) influenced immune responses in the heart and spleen, as well as cardiac remodeling, following MI. The study revealed a reduced ratio of proinflammatory to anti-inflammatory macrophages in the myocardium and lower levels of TNF-α in the hearts and spleens of SHRs treated with a cholinesterase inhibitor [124]. 

In a separate investigation involving SHRs expressing the human CRP transgene (SHRs-CRP), animals experienced recurrent and prolonged ventricular tachyarrhythmias following MI. Interestingly, this led to a notable reduction in infarct size in SHRs-CRP compared to SHRs. Acute ischemia notably elevated the levels of several cardioprotective molecules in SHRs-CRP [125].

In summary, the inflammatory response is crucial to repairing the infarcted heart, wherein immune cells play a fine-tuned dual role in injury and protection. However, the presence of metabolic risk factors has been shown to interfere with and deregulate both local and systemic inflammatory reactions, interfering with proper cardiac healing and increasing the risk of adverse left ventricular remodeling and eventual heart failure. Consequently, prioritizing the management of cardiometabolic risk factors may influence cardiac repair following MI.

## 6. Impact of Lifestyle Changes and Therapeutic Approaches

Adopting healthy dietary habits is essential for reducing cardiometabolic risks [126]. Several epidemiological studies support an inverse association between adherence to healthy dietary patterns, such as the Mediterranean diet (abundant in minimally processed plant-based foods, rich in monounsaturated fat from extra virgin olive oil, and low in saturated fat, meats, and dairy products [127,128]), and cardiometabolic risk. In this context, the PREDIMED trial evidenced that participants following a Mediterranean diet supplemented with either extra virgin olive oil or nuts were able to reverse their disrupted metabolic state. In fact, participants in the group receiving olive oil supplementation showed a significant decline in central obesity and high fasting glucose [129]. Furthermore, individuals with metabolic syndrome experienced decreased systemic inflammatory markers (such as IL-6, IL-7, IL-18, and high-sensitive CRP) following the consumption of an olive oil-supplemented diet [130]. Among STEMI patients, the levels of high-sensitive CRP were lower in those with higher adhesion to the Mediterranean diet [131]. While the anti-inflammatory advantages of the Mediterranean diet are often linked to its abundance of antioxidants, the specific mechanisms underlying this phenomenon are yet to be fully elucidated. Another area of interest concerning the impact of diet on the MI-related inflammatory response involves the microbiota. Studies conducted in vitro, in vivo, and in humans have suggested that a healthy microbiome regulates immune responses and lessens MI size. As such, administering a combination of two probiotics (*Lactobacillus helveticus* and *Bifidobacterium longum*) before and after MI has been shown to decrease inflammatory cytokine release and apoptosis execution [132].

On the other hand, regular exercise has also been shown to improve glycemic control in patients with T2D [133], and aerobic and resistance exercise training reduces systolic and diastolic blood pressure to a comparable level to that achieved by antihypertensive treatment [134]. The Dietary Approaches to Stop Hypertension (DASH) diet, which emphasizes fruits, vegetables, and low-fat dairy products, has been shown to lower blood pressure [135]. In addition, different classes of medications, like angiotensin-converting enzyme (ACE) inhibitors and angiotensin-II receptor blockers (ARBs), are used to control hypertension and are associated with cardioprotective properties. This is because they may mitigate the adverse effects of angiotensin II during the onset of MI. A meta-analysis that compared the clinical outcomes of both drugs in patients with MI showed that these medications have similar results across a broad spectrum of MI patients, reinforcing their roles in post-MI treatment [136]. Regular exercise has also been shown to benefit lipid profiles (higher HDL-cholesterol and lower LDL-cholesterol and TGs) [137]. In the context of MI, the post- and pre-exercise effects on cardiac remodeling and function have been investigated in mice. Thus, while physical activity did not affect MI-induced cardiac hypertrophy in sedentary mice compared to those engaging in voluntary exercise on a running wheel, the exercised group exhibited decreased collagen content in the developing scar. In addition, transcript levels of proinflammatory cytokines (TNF-α, IL-6, and IL-1β) correlated positively with infarct size and collagen mRNA expression in sedentary mice, whereas this correlation was attenuated or even absent in the trained group [138]. However, more data are needed to support these findings.

Sodium–glucose co-transporter 2 inhibitors (SGLT2 inhibitors or SGLT2-Is) have garnered significant clinical interest in both diabetic and non-diabetic patients. A clinical trial involving patients with T2D and MI treated with SGLT2 inhibitors showed a notable reduction in inflammatory response and smaller infarct size compared to those treated with other oral anti-diabetic agents [139]. Given the complexities of managing the ischemic diabetic heart, controlling the inflammatory response through SGLT2 inhibitors may become a promising therapeutic approach.

Emerging data have supported the benefits of reducing TG levels in protecting against the occurrence of all fatal or non-fatal cardiovascular events among patients at high cardiovascular risk [140]. The recent REDUCE-IT trial demonstrated that the administration of high-dose icosapent ethyl (IPE) [an ethyl ester of eicosapentaenoic acid (EPA; ω3 fatty acid)] resulted in a significant reduction in TG levels, which was accompanied by a 25% reduction in cardiovascular events [140]. The EVAPORATE trial provided a plausible mechanistic understanding to support these findings. As such, IPE administration was associated with atherosclerotic plaque regression and a less vulnerable plaque phenotype (less fibroadipose proinflammatory plaques) [141]. Regarding hypercholesterolemia, statins serve as the cornerstone treatment, potentially lowering LDL-cholesterol levels by as much as 50% [142]. Beyond lipid-lowering, high-dose statins have been shown to exert cardioprotective effects by modulating local and systemic inflammatory responses. We previously demonstrated in pigs subjected to I/R that intravenous administration of atorvastatin limits the MI-induced inflammatory response by preventing both MCP-1 mRNA and protein upregulation in the ischemic cardiac zone, reducing neutrophil recruitment by around 50%, lessening monocyte peripheral blood mononuclear cell activation, and acutely enhancing plasma TNF-α levels [higher survivor activating factor enhancement (SAFE) pathways activation] as compared to control pigs [143]. In line with these findings, patients with MI treated with atorvastatin have shown a reduction in serum inflammatory markers [144]. Moreover, a recent study demonstrated that the adoptive transfer of DCs cultured with the supernatant from infarcted mice plus atorvastatin alleviated post-infarction cardiomyocyte apoptosis and myocardial fibrosis, in association with decreased inflammatory cell infiltration and inhibited oxidative stress, likely by suppressing TLR4/NFκB activation after MI [145]. Pitavastatin, used with a nanoparticle-mediated delivery method in CD11b^+^ monocytes/macrophages, has also led to a reduction in monocytes/macrophages in the heart by inhibiting monocyte mobilization from the spleen after MI. These results suggest that the inhibition of monocyte mobilization from the bone marrow is one of the major mechanisms by which this statin attenuates post-infarct left ventricle remodeling [146]. Moreover, in a rat model of MI, intramyocardial injection of mesenchymal stem cell-derived EVs, pre-treated with atorvastatin, not only restricted macrophage infiltration but also delivered EV miR-139-3p to macrophages, promoting their transition to an anti-inflammatory phenotype [147]. Whether miR-139-3p can affect other cardiac cells remains to be addressed. Other lipid-lowering agents, such as proprotein convertase subtilisin/kesin 9 (PCSK9) inhibitors, have also been shown to modulate the inflammatory response. PCSK9 is involved in NFκB signaling, leading to an enhanced secretion of TNF-α, IL-6, and IL-1β by macrophages and worsening hypoxia–reoxygenation-induced injury in cardiomyocytes [148]. Furthermore, PCSK9 has also been shown to cooperate with LDL receptor-related protein 5 (LRP5) in TLR4/NFκB signaling. Reduced TLR4 protein expression levels and decreased nuclear NFκB translocation were observed in PCSK9-silenced cells after lipid loading, indicating the downregulation of the TLR4/NFκB signaling pathway and demonstrating the ability of LRP5 to favor macrophage lipid uptake and form a complex with PCSK9, which, in turn, upregulates TLR4/NFκB, promoting inflammation [149]. In a recent study, patients treated with PCSK9 inhibitors exhibited reduced expression of proinflammatory proteins compared to those treated with other lipid-lowering therapies, despite having comparable levels of high-sensitivity CRP [150]. However, the authors did not explore the potential influence of HDL-cholesterol and TG levels on these findings. Furthermore, the Canakinumab Antiinflammatory Thrombosis Outcomes Study (CANTOS) showed that blocking IL-1β with the monoclonal antibody canakinumab improved cardiovascular outcomes in high-risk patients without altering lipid levels. However, patients treated with canakinumab experienced higher rates of fatal infections, neutropenia, and thrombocytopenia compared to those who received a placebo [151]. LDL-cholesterol-lowering therapies do not exhibit these adverse effects, highlighting the importance of cautious administration of anti-inflammatory agents.

## 7. Conclusions and Future Perspectives

Inflammation is a key factor in the healing process after MI and is also associated with metabolic disorders. Unraveling the mechanisms through which metabolic risk factors influence the inflammatory response may facilitate the development of therapeutic approaches tailored to the metabolic condition of the patient (precision medicine). Likewise, lifestyle changes, particularly focusing on diet and physical activity, have shown significant effects on metabolic factors such as dyslipidemia, diabetes, and hypertension. It is crucial to delve deeper into how these lifestyle adjustments affect the inflammatory response following MI. Such insights could pave the way for the adoption of more efficient approaches to handle post-MI inflammation and enhance cardiovascular health outcomes holistically.

## References

[1] Salari N., Morddarvanjoghi F., Abdolmaleki A., Rasoulpoor S., Khaleghi A.A., Hezarkhani L.A., Shohaimi S., Mohammadi M. (2023). The Global Prevalence of Myocardial Infarction: A Systematic Review and Meta-Analysis. BMC Cardiovasc. Disord..

[2] Vilahur G., Badimon J.J., Bugiardini R., Badimon L. (2014). Perspectives: The Burden of Cardiovascular Risk Factors and Coronary Heart Disease in Europe and Worldwide. Eur. Heart J. Suppl..

[3] Frangogiannis N.G. (2014). The Inflammatory Response in Myocardial Injury, Repair, and Remodelling. Nat. Rev. Cardiol..

[4] Vilahur G., Juan-Babot O., Peña E., Oñate B., Casaní L., Badimon L. (2011). Molecular and Cellular Mechanisms Involved in Cardiac Remodeling after Acute Myocardial Infarction. JMCC.

[5] Andreadou I., Cabrera-Fuentes H.A., Devaux Y., Frangogiannis N.G., Frantz S., Guzik T., Liehn E.A., Gomes C.P.C., Schulz R., Hausenloy D.J. (2019). Immune Cells as Targets for Cardioprotection: New Players and Novel Therapeutic Opportunities. Cardiovasc. Res..

[6] Jenča D., Melenovský V., Stehlik J., Staněk V., Kettner J., Josef K., Adámková V., Wohlfahrt P. (2021). Heart Failure after Myocardial Infarction: Incidence and Predictors. ESC Heart Fail..

[7] Gerdts E., Regitz-Zagrosek V. (2019). Sex Differences in Cardiometabolic Disorders. Nat. Med..

[8] Teo K.K., Rafiq T. (2021). Cardiovascular Risk Factors and Prevention: A Perspective from Developing Countries. Can. J. Cardiol..

[9] Vilahur G., Casani L., Juan-Babot O., Guerra J.M., Badimon L. (2012). Infiltrated Cardiac Lipids Impair Myofibroblast-Induced Healing of the Myocardial Scar Post-Myocardial Infarction. Atherosclerosis.

[10] Andreadou I., Schulz R., Badimon L., Adameová A., Kleinbongard P., Lecour S., Nikolaou P., Falcão-Pires I., Vilahur G., Woudberg N. (2020). Hyperlipidaemia and Cardioprotection: Animal Models for Translational Studies. Br. J. Pharmacol..

[11] Frangogiannis N. (2008). The Immune System and Cardiac Repair. Pharmacol. Res..

[12] Anzai A., Ko S., Fukuda K. (2022). Immune and Inflammatory Networks in Myocardial Infarction: Current Research and Its Potential Implications for the Clinic. IJMS.

[13] Foglio E., Pellegrini L., Russo M.A., Limana F. (2022). HMGB1-Mediated Activation of the Inflammatory-Reparative Response Following Myocardial Infarction. Cells.

[14] Jaén R.I., Val-Blasco A., Prieto P., Gil-Fernández M., Smani T., López-Sendón J.L., Delgado C., Boscá L., Fernández-Velasco M. (2020). Innate Immune Receptors, Key Actors in Cardiovascular Diseases. JACC Basic Transl. Sci..

[15] Vilahur G., Badimon L. (2014). Ischemia/Reperfusion Activates Myocardial Innate Immune Response: The Key Role of the Toll-like Receptor. Front. Physiol..

[16] Kim S.-C., Ghanem A., Stapel H., Tiemann K., Knuefermann P., Hoeft A., Meyer R., Grohé C., Knowlton A.A., Baumgarten G. (2007). Toll-like Receptor 4 Deficiency: Smaller Infarcts, but Nogain in Function. BMC Physiol..

[17] Riad A., Jäger S., Sobirey M., Escher F., Yaulema-Riss A., Westermann D., Karatas A., Heimesaat M.M., Bereswill S., Dragun D. (2008). Toll-Like Receptor-4 Modulates Survival by Induction of Left Ventricular Remodeling after Myocardial Infarction in Mice. J. Immun..

[18] Bucciarelli L.G., Ananthakrishnan R., Hwang Y.C., Kaneko M., Song F., Sell D.R., Strauch C., Monnier V.M., Yan S.F., Schmidt A.M. (2008). RAGE and Modulation of Ischemic Injury in the Diabetic Myocardium. Diabetes.

[19] Yan S.F., Ramasamy R., Schmidt A.M. (2009). The Receptor for Advanced Glycation Endproducts (RAGE) and Cardiovascular Disease. Expert Rev. Mol. Med..

[20] Hohensinner P.J., Niessner A., Huber K., Weyand C.M., Wojta J. (2011). Inflammation and Cardiac Outcome. Curr. Opin. Infect. Dis..

[21] Chen B., Frangogiannis N.G. (2021). Chemokines in Myocardial Infarction. J. Cardiovasc. Trans. Res..

[22] Torp M.-K., Vaage J., Stensløkken K.-O. (2023). Mitochondria-Derived Damage-Associated Molecular Patterns and Inflammation in the Ischemic-Reperfused Heart. Acta Physiol..

[23] Longnus S.L., Rutishauser N., Gillespie M.N., Reichlin T., Carrel T.P., Sanz M.N. (2021). Mitochondrial Damage-Associated Molecular Patterns as Potential Biomarkers in DCD Heart Transplantation: Lessons from Myocardial Infarction and Cardiac Arrest. Transplant. Direct.

[24] Barbalata T., Scarlatescu A.I., Sanda G.M., Toma L., Stancu C.S., Dorobantu M., Micheu M.M., Sima A.V., Niculescu L.S. (2022). Mitochondrial DNA Together with miR-142-3p in Plasma Can Predict Unfavorable Outcomes in Patients after Acute Myocardial Infarction. IJMS.

[25] Peng N., Guo L., Wei Z., Wang X., Zhao L., Kang L., Wang K., Zhou W., Cheng S., Yin S. (2024). Platelet Mitochondrial DNA Methylation: A Novel Biomarker for Myocardial Infarction—A Preliminary Study. Int. J. Cardiol..

[26] Frangogiannis N.G. (2014). The Immune System and the Remodeling Infarcted Heart: Cell Biological Insights and Therapeutic Opportunities. J. Cardiovasc. Pharmacol..

[27] Chakraborti T., Mandal A., Mandal M., Das S., Chakraborti S. (2000). Complement Activation in Heart Diseases: Role of Oxidants. Cell. Signal..

[28] Toldo S., Abbate A. (2023). The Role of the NLRP3 Inflammasome and Pyroptosis in Cardiovascular Diseases. Nat. Rev. Cardiol..

[29] Wu J., Chen S., Liu Y., Liu Z., Wang D., Cheng Y. (2020). Therapeutic Perspectives of Heat Shock Proteins and Their Protein-Protein Interactions in Myocardial Infarction. Pharmacol. Res..

[30] Chen Z., He L., Li L., Chen L. (2018). The P2X7 Purinergic Receptor: An Emerging Therapeutic Target in Cardiovascular Diseases. Clin. Chim. Acta.

[31] Yin J., Wang Y., Hu H., Li X., Xue M., Cheng W., Wang Y., Li X., Yang N., Shi Y. (2017). P2X_7_ Receptor Inhibition Attenuated Sympathetic Nerve Sprouting after Myocardial Infarction via the NLRP3/IL-1β Pathway. J. Cell. Mol. Med..

[32] Zapata-Martínez L., Águila S., De Los Reyes-García A.M., Carrillo-Tornel S., Lozano M.L., González-Conejero R., Martínez C. (2023). Inflammatory microRNAs in Cardiovascular Pathology: Another Brick in the Wall. Front. Immunol..

[33] Varzideh F., Kansakar U., Donkor K., Wilson S., Jankauskas S.S., Mone P., Wang X., Lombardi A., Santulli G. (2022). Cardiac Remodeling after Myocardial Infarction: Functional Contribution of microRNAs to Inflammation and Fibrosis. Front. Cardiovasc. Med..

[34] Sessa F., Salerno M., Esposito M., Cocimano G., Pisanelli D., Malik A., Khan A.A., Pomara C. (2023). New Insight into Mechanisms of Cardiovascular Diseases: An Integrative Analysis Approach to Identify TheranoMiRNAs. IJMS.

[35] Sessa F., Salerno M., Esposito M., Cocimano G., Pomara C. (2023). miRNA Dysregulation in Cardiovascular Diseases: Current Opinion and Future Perspectives. IJMS.

[36] Han W., Wang W., Wang Q., Maduray K., Hao L., Zhong J. (2024). A Review on Regulation of DNA Methylation during Post-Myocardial Infarction. Front. Pharmacol..

[37] Fang L., Moore X.-L., Dart A.M., Wang L.-M. (2015). Systemic Inflammatory Response Following Acute Myocardial Infarction. J. Geriatr. Cardiol..

[38] Vilahur G., Hernández-Vera R., Molins B., Casaní L., Duran X., Padró T., Badimon L. (2009). Short-term Myocardial Ischemia Induces Cardiac Modified C-reactive Protein Expression and Proinflammatory Gene (Cyclo-oxygenase-2, Monocyte Chemoattractant Protein-1, and Tissue Factor) Upregulation in Peripheral Blood Mononuclear Cells. J. Thromb. Haemost..

[39] Morrison S.J., Scadden D.T. (2014). The Bone Marrow Niche for Haematopoietic Stem Cells. Nature.

[40] Poller W.C., Nahrendorf M., Swirski F.K. (2020). Hematopoiesis and Cardiovascular Disease. Circ. Res..

[41] Sager H.B., Heidt T., Hulsmans M., Dutta P., Courties G., Sebas M., Wojtkiewicz G.R., Tricot B., Iwamoto Y., Sun Y. (2015). Targeting Interleukin-1β Reduces Leukocyte Production after Acute Myocardial Infarction. Circulation.

[42] Crane G.M., Jeffery E., Morrison S.J. (2017). Adult Haematopoietic Stem Cell Niches. Nat. Rev. Immunol..

[43] Anzai A., Choi J.L., He S., Fenn A.M., Nairz M., Rattik S., McAlpine C.S., Mindur J.E., Chan C.T., Iwamoto Y. (2017). The Infarcted Myocardium Solicits GM-CSF for the Detrimental Oversupply of Inflammatory Leukocytes. J. Exp. Med..

[44] Seung H., Wrobel J., Wadle C., Bühler T., Chiang D., Rettkowski J., Cabezas-Wallscheid N., Hechler B., Stachon P., Maier A. (2022). P2Y_12_-dependent Activation of Hematopoietic Stem and Progenitor Cells Promotes Emergency Hematopoiesis after Myocardial Infarction. Basic. Res. Cardiol..

[45] Rohde D., Vandoorne K., Lee I.-H., Grune J., Zhang S., McAlpine C.S., Schloss M.J., Nayar R., Courties G., Frodermann V. (2021). Bone Marrow Endothelial Dysfunction Promotes Myeloid Cell Expansion in Cardiovascular Disease. Nat. Cardiovasc. Res..

[46] Maryanovich M., Takeishi S., Frenette P.S. (2018). Neural Regulation of Bone and Bone Marrow. Cold Spring Harb. Perspect. Med..

[47] Nahrendorf M. (2018). Myeloid Cell Contributions to Cardiovascular Health and Disease. Nat. Med..

[48] Nakatani T., Sugiyama T., Omatsu Y., Watanabe H., Kondoh G., Nagasawa T. (2023). Ebf3^+^ Niche-Derived CXCL12 Is Required for the Localization and Maintenance of Hematopoietic Stem Cells. Nat. Commun..

[49] Dutta P., Courties G., Wei Y., Leuschner F., Gorbatov R., Robbins C.S., Iwamoto Y., Thompson B., Carlson A.L., Heidt T. (2012). Myocardial Infarction Accelerates Atherosclerosis. Nature.

[50] Comazzetto S., Shen B., Morrison S.J. (2021). Niches That Regulate Stem Cells and Hematopoiesis in Adult Bone Marrow. Dev. Cell.

[51] Fernández-García V., González-Ramos S., Martín-Sanz P., Castrillo A., Boscá L. (2020). Contribution of Extramedullary Hematopoiesis to Atherosclerosis. The Spleen as a Neglected Hub of Inflammatory Cells. Front. Immunol..

[52] Swirski F.K., Nahrendorf M., Etzrodt M., Wildgruber M., Cortez-Retamozo V., Panizzi P., Figueiredo J.-L., Kohler R.H., Chudnovskiy A., Waterman P. (2009). Identification of Splenic Reservoir Monocytes and Their Deployment to Inflammatory Sites. Science.

[53] Leuschner F., Panizzi P., Chico-Calero I., Lee W.W., Ueno T., Cortez-Retamozo V., Waterman P., Gorbatov R., Marinelli B., Iwamoto Y. (2010). Angiotensin-Converting Enzyme Inhibition Prevents the Release of Monocytes from Their Splenic Reservoir in Mice with Myocardial Infarction. Circ. Res..

[54] Leuschner F., Rauch P.J., Ueno T., Gorbatov R., Marinelli B., Lee W.W., Dutta P., Wei Y., Robbins C., Iwamoto Y. (2012). Rapid Monocyte Kinetics in Acute Myocardial Infarction Are Sustained by Extramedullary Monocytopoiesis. J. Exp. Med..

[55] Dutta P., Hoyer F.F., Grigoryeva L.S., Sager H.B., Leuschner F., Courties G., Borodovsky A., Novobrantseva T., Ruda V.M., Fitzgerald K. (2015). Macrophages Retain Hematopoietic Stem Cells in the Spleen via VCAM-1. J. Exp. Med..

[56] Tawakol A., Ishai A., Takx R.A., Figueroa A.L., Ali A., Kaiser Y., Truong Q.A., Solomon C.J., Calcagno C., Mani V. (2017). Relation between Resting Amygdalar Activity and Cardiovascular Events: A Longitudinal and Cohort Study. Lancet.

[57] Ma Y., Yabluchanskiy A., Lindsey M.L. (2013). Neutrophil Roles in Left Ventricular Remodeling Following Myocardial Infarction. Fibrogenesis Tissue Repair.

[58] Liew P.X., Kubes P. (2019). The Neutrophil’s Role during Health and Disease. Physiol. Rev..

[59] Peterson E.A., Sun J., Wang J. (2022). Leukocyte-Mediated Cardiac Repair after Myocardial Infarction in Non-Regenerative vs. Regenerative Systems. JCDD.

[60] Ma Y. (2021). Role of Neutrophils in Cardiac Injury and Repair Following Myocardial Infarction. Cells.

[61] Ma Y., Yang X., Chatterjee V., Meegan J.E., Beard R.S., Yuan S.Y. (2019). Role of Neutrophil Extracellular Traps and Vesicles in Regulating Vascular Endothelial Permeability. Front. Immunol..

[62] Jung K., Kim P., Leuschner F., Gorbatov R., Kim J.K., Ueno T., Nahrendorf M., Yun S.H. (2013). Endoscopic Time-Lapse Imaging of Immune Cells in Infarcted Mouse Hearts. Circ. Res..

[63] Stierschneider A., Wiesner C. (2023). Shedding Light on the Molecular and Regulatory Mechanisms of TLR4 Signaling in Endothelial Cells under Physiological and Inflamed Conditions. Front. Immunol..

[64] Medrano-Bosch M., Simón-Codina B., Jiménez W., Edelman E.R., Melgar-Lesmes P. (2023). Monocyte-Endothelial Cell Interactions in Vascular and Tissue Remodeling. Front. Immunol..

[65] Deshmane S.L., Kremlev S., Amini S., Sawaya B.E. (2009). Monocyte Chemoattractant Protein-1 (MCP-1): An Overview. J. Interferon Cytokine Res..

[66] Nahrendorf M., Swirski F.K., Aikawa E., Stangenberg L., Wurdinger T., Figueiredo J.-L., Libby P., Weissleder R., Pittet M.J. (2007). The Healing Myocardium Sequentially Mobilizes Two Monocyte Subsets with Divergent and Complementary Functions. J. Exp. Med..

[67] Lafuse W.P., Wozniak D.J., Rajaram M.V.S. (2020). Role of Cardiac Macrophages on Cardiac Inflammation, Fibrosis and Tissue Repair. Cells.

[68] Bajpai G., Schneider C., Wong N., Bredemeyer A., Hulsmans M., Nahrendorf M., Epelman S., Kreisel D., Liu Y., Itoh A. (2018). The Human Heart Contains Distinct Macrophage Subsets with Divergent Origins and Functions. Nat. Med..

[69] Bajpai G., Bredemeyer A., Li W., Zaitsev K., Koenig A., Lokshina I., Mohan J., Ivey B., Hsiao H.-M., Weinheimer C. (2019). Tissue Resident CCR2^−^ and CCR2^+^ Cardiac Macrophages Differentially Orchestrate Monocyte Recruitment and Fate Specification Following Myocardial Injury. Circ. Res..

[70] Chen B., Brickshawana A., Frangogiannis N.G. (2019). The Functional Heterogeneity of Resident Cardiac Macrophages in Myocardial Injury: CCR2^+^ Cells Promote Inflammation, Whereas CCR2^−^ Cells Protect. Circ. Res..

[71] Hitscherich P., Lee E.J. (2021). Crosstalk between Cardiac Cells and Macrophages Postmyocardial Infarction: Insights from In Vitro Studies. Tissue Eng. Part B Rev..

[72] Kologrivova I., Shtatolkina M., Suslova T., Ryabov V. (2021). Cells of the Immune System in Cardiac Remodeling: Main Players in Resolution of Inflammation and Repair after Myocardial Infarction. Front. Immunol..

[73] Wan E., Yeap X.Y., Dehn S., Terry R., Novak M., Zhang S., Iwata S., Han X., Homma S., Drosatos K. (2013). Enhanced Efferocytosis of Apoptotic Cardiomyocytes through Myeloid-Epithelial-Reproductive Tyrosine Kinase Links Acute Inflammation Resolution to Cardiac Repair after Infarction. Circ. Res..

[74] Frangogiannis N.G. (2015). Inflammation in Cardiac Injury, Repair and Regeneration. Curr. Opin. Cardiol..

[75] Shiraishi M., Shintani Y., Shintani Y., Ishida H., Saba R., Yamaguchi A., Adachi H., Yashiro K., Suzuki K. (2016). Alternatively Activated Macrophages Determine Repair of the Infarcted Adult Murine Heart. J. Clin. Investig..

[76] Dick S.A., Macklin J.A., Nejat S., Momen A., Clemente-Casares X., Althagafi M.G., Chen J., Kantores C., Hosseinzadeh S., Aronoff L. (2019). Self-Renewing Resident Cardiac Macrophages Limit Adverse Remodeling Following Myocardial Infarction. Nat. Immunol..

[77] Wang L., Zhang Y., Yu M., Yuan W. (2022). Identification of Hub Genes in the Remodeling of Non-Infarcted Myocardium Following Acute Myocardial Infarction. JCDD.

[78] Jiang Y., Yu W., Hu T., Peng H., Hu F., Yuan Y., Liu X., Lai S., Zhou J., Dong X. (2024). Unveiling Macrophage Diversity in Myocardial Ischemia-Reperfusion Injury: Identification of a Distinct Lipid-Associated Macrophage Subset. Front. Immunol..

[79] Lee J.S., Jeong S.-J., Kim S., Chalifour L., Yun T.J., Miah M.A., Li B., Majdoubi A., Sabourin A., Keler T. (2018). Conventional Dendritic Cells Impair Recovery after Myocardial Infarction. J. Immunol..

[80] Venugopal H., Hanna A., Humeres C., Frangogiannis N.G. (2022). Properties and Functions of Fibroblasts and Myofibroblasts in Myocardial Infarction. Cells.

[81] Shinde A.V., Frangogiannis N.G. (2014). Fibroblasts in Myocardial Infarction: A Role in Inflammation and Repair. J. Mol. Cell. Cardiol..

[82] Fu X., Khalil H., Kanisicak O., Boyer J.G., Vagnozzi R.J., Maliken B.D., Sargent M.A., Prasad V., Valiente-Alandi I., Blaxall B.C. (2018). Specialized Fibroblast Differentiated States Underlie Scar Formation in the Infarcted Mouse Heart. J. Clin. Investig..

[83] Hofmann U., Frantz S. (2016). Role of T-Cells in Myocardial Infarction. Eur. Heart J..

[84] Yan X., Shichita T., Katsumata Y., Matsuhashi T., Ito H., Ito K., Anzai A., Endo J., Tamura Y., Kimura K. (2012). Deleterious Effect of the IL-23/IL-17A Axis and γδT Cells on Left Ventricular Remodeling after Myocardial Infarction. JAHA.

[85] Liao Y.-H., Cheng X. (2006). Autoimmunity in Myocardial Infarction. Int. J. Cardiol..

[86] Ilatovskaya D.V., Pitts C., Clayton J., Domondon M., Troncoso M., Pippin S., DeLeon-Pennell K.Y. (2019). CD8 ^+^ T-Cells Negatively Regulate Inflammation Post-Myocardial Infarction. Am. J. Physiol. Heart Circ. Physiol..

[87] Hofmann U., Frantz S. (2015). Role of Lymphocytes in Myocardial Injury, Healing, and Remodeling after Myocardial Infarction. Circ. Res..

[88] Yu Y.-R.A., O’Koren E.G., Hotten D.F., Kan M.J., Kopin D., Nelson E.R., Que L., Gunn M.D. (2016). A Protocol for the Comprehensive Flow Cytometric Analysis of Immune Cells in Normal and Inflamed Murine Non-Lymphoid Tissues. PLoS ONE.

[89] Haas M.S., Alicot E.M., Schuerpf F., Chiu I., Li J., Moore F.D., Carroll M.C. (2010). Blockade of Self-Reactive IgM Significantly Reduces Injury in a Murine Model of Acute Myocardial Infarction. Cardiovasc. Res..

[90] Zouggari Y., Ait-Oufella H., Bonnin P., Simon T., Sage A.P., Guérin C., Vilar J., Caligiuri G., Tsiantoulas D., Laurans L. (2013). B Lymphocytes Trigger Monocyte Mobilization and Impair Heart Function after Acute Myocardial Infarction. Nat. Med..

[91] Wu L., Dalal R., Cao C.D., Postoak J.L., Yang G., Zhang Q., Wang Z., Lal H., Van Kaer L. (2019). IL-10–Producing B Cells Are Enriched in Murine Pericardial Adipose Tissues and Ameliorate the Outcome of Acute Myocardial Infarction. Proc. Natl. Acad. Sci. USA.

[92] Pluijmert N.J., Den Haan M.C., Van Zuylen V.L., Steendijk P., De Boer H.C., Van Zonneveld A.J., Fibbe W.E., Schalij M.J., Quax P.H.A., Atsma D.E. (2019). Hypercholesterolemia Affects Cardiac Function, Infarct Size and Inflammation in APOE*3-Leiden Mice Following Myocardial Ischemia-Reperfusion Injury. PLoS ONE.

[93] Mączewski M., Mączewska J. (2006). Hypercholesterolemia Exacerbates Ventricular Remodeling in the Rat Model of Myocardial Infarction. J. Card. Fail..

[94] Andreadou I., Mitakou S., Paraschos S., Efentakis P., Magiatis P., Kaklamanis L., Halabalaki M., Skaltsounis L., Iliodromitis E.K. (2016). “*Pistacia Lentiscus* L.” Reduces the Infarct Size in Normal Fed Anesthetized Rabbits and Possess Antiatheromatic and Hypolipidemic Activity in Cholesterol Fed Rabbits. Phytomedicine.

[95] Osipov R.M., Bianchi C., Feng J., Clements R.T., Liu Y., Robich M.P., Glazer H.P., Sodha N.R., Sellke F.W. (2009). Effect of Hypercholesterolemia on Myocardial Necrosis and Apoptosis in the Setting of Ischemia-Reperfusion. Circulation.

[96] Zhao J.-L., Yang Y.-J., You S.-J., Cui C.-J., Gao R.-L. (2007). Different Effects of Postconditioning on Myocardial No-Reflow in the Normal and Hypercholesterolemic Mini-Swines. Microvasc. Res..

[97] Ferdinandy P., Andreadou I., Baxter G.F., Bøtker H.E., Davidson S.M., Dobrev D., Gersh B.J., Heusch G., Lecour S., Ruiz-Meana M. (2023). Interaction of Cardiovascular Nonmodifiable Risk Factors, Comorbidities and Comedications with Ischemia/Reperfusion Injury and Cardioprotection by Pharmacological Treatments and Ischemic Conditioning. Pharmacol. Rev..

[98] Ben-Aicha S., Badimon L., Vilahur G. (2020). Advances in HDL: Much More than Lipid Transporters. IJMS.

[99] Choi B., Vilahur G., Yadegar D., Viles-Gonzalez J., Badimon J. (2006). The Role of High-Density Lipoprotein Cholesterol in the Prevention and Possible Treatment of Cardiovascular Diseases. CMM.

[100] Ben-Aicha S., Escate R., Casaní L., Padró T., Peña E., Arderiu G., Mendieta G., Badimón L., Vilahur G. (2020). High-Density Lipoprotein Remodelled in Hypercholesterolaemic Blood Induce Epigenetically Driven down-Regulation of Endothelial HIF-1α Expression in a Preclinical Animal Model. Cardiovasc. Res..

[101] Ben-Aicha S., Casaní L., Muñoz-García N., Joan-Babot O., Peña E., Aržanauskaitė M., Gutierrez M., Mendieta G., Padró T., Badimon L. (2020). HDL (High-Density Lipoprotein) Remodeling and Magnetic Resonance Imaging–Assessed Atherosclerotic Plaque Burden: Study in a Preclinical Experimental Model. ATVB.

[102] Padró T., Cubedo J., Camino S., Béjar M.T., Ben-Aicha S., Mendieta G., Escolà-Gil J.C., Escate R., Gutiérrez M., Casani L. (2017). Detrimental Effect of Hypercholesterolemia on High-Density Lipoprotein Particle Remodeling in Pigs. J. Am. Coll. Cardiol..

[103] Schoch L., Sutelman P., Suades R., Casani L., Padro T., Badimon L., Vilahur G. (2022). Hypercholesterolemia-Induced HDL Dysfunction Can Be Reversed: The Impact of Diet and Statin Treatment in a Preclinical Animal Model. IJMS.

[104] Nordestgaard B.G., Benn M., Schnohr P., Tybjærg-Hansen A. (2007). Nonfasting Triglycerides and Risk of Myocardial Infarction, Ischemic Heart Disease, and Death in Men and Women. JAMA.

[105] Ting H.J., Stice J.P., Schaff U.Y., Hui D.Y., Rutledge J.C., Knowlton A.A., Passerini A.G., Simon S.I. (2007). Triglyceride-Rich Lipoproteins Prime Aortic Endothelium for an Enhanced Inflammatory Response to Tumor Necrosis Factor-α. Circ. Res..

[106] Farnier M., Zeller M., Masson D., Cottin Y. (2021). Triglycerides and Risk of Atherosclerotic Cardiovascular Disease: An Update. Arch. Cardiovasc. Dis..

[107] Miller M., Stone N.J., Ballantyne C., Bittner V., Criqui M.H., Ginsberg H.N., Goldberg A.C., Howard W.J., Jacobson M.S., Kris-Etherton P.M. (2011). Triglycerides and Cardiovascular Disease: A Scientific Statement from the American Heart Association. Circulation.

[108] Doi T., Langsted A., Nordestgaard B.G. (2023). Dual Elevated Remnant Cholesterol and C-Reactive Protein in Myocardial Infarction, Atherosclerotic Cardiovascular Disease, and Mortality. Atherosclerosis.

[109] Mastrocola R., Collino M., Penna C., Nigro D., Chiazza F., Fracasso V., Tullio F., Alloatti G., Pagliaro P., Aragno M. (2016). Maladaptive Modulations of NLRP3 Inflammasome and Cardioprotective Pathways Are Involved in Diet-Induced Exacerbation of Myocardial Ischemia/Reperfusion Injury in Mice. Oxidative Med. Cell. Longev..

[110] Padró T., Vilahur G., Badimon L. (2018). Dyslipidemias and Microcirculation. Curr. Pharm. Des..

[111] Babbar L., Mahadevan N., Balakumar P. (2013). Fenofibrate Attenuates Impaired Ischemic Preconditioning-Mediated Cardioprotection in the Fructose-Fed Hypertriglyceridemic Rat Heart. Naunyn-Schmiedeberg’s Arch. Pharmacol..

[112] Khalid M., Petroianu G., Adem A. (2022). Advanced Glycation End Products and Diabetes Mellitus: Mechanisms and Perspectives. Biomolecules.

[113] Bansal S., Burman A., Tripathi A.K. (2023). Advanced Glycation End Products: Key Mediator and Therapeutic Target of Cardiovascular Complications in Diabetes. World J. Diabetes.

[114] Uchasova E., Gruzdeva, Belik E., Shurygina, Barbarash O., Dyleva Y. (2013). Plasminogen Activator Inhibitor-1, Free Fatty Acids, and Insulin Resistance in Patients with Myocardial Infarction. DMSO.

[115] Madrazo J.A., Kelly D.P. (2008). The PPAR Trio: Regulators of Myocardial Energy Metabolism in Health and Disease. J. Cell. Mol. Med..

[116] Battiprolu P.K., Hojayev B., Jiang N., Wang Z.V., Luo X., Iglewski M., Shelton J.M., Gerard R.D., Rothermel B.A., Gillette T.G. (2012). Metabolic Stress–Induced Activation of FoxO1 Triggers Diabetic Cardiomyopathy in Mice. J. Clin. Investig..

[117] Lee H.-M., Kim J.-J., Kim H.J., Shong M., Ku B.J., Jo E.-K. (2013). Upregulated NLRP3 Inflammasome Activation in Patients with Type 2 Diabetes. Diabetes.

[118] Luo B., Li B., Wang W., Liu X., Xia Y., Zhang C., Zhang M., Zhang Y., An F. (2014). NLRP3 Gene Silencing Ameliorates Diabetic Cardiomyopathy in a Type 2 Diabetes Rat Model. PLoS ONE.

[119] Tocci G., Figliuzzi I., Presta V., Miceli F., Citoni B., Coluccia R., Musumeci M.B., Ferrucci A., Volpe M. (2018). Therapeutic Approach to Hypertension Urgencies and Emergencies during Acute Coronary Syndrome. High. Blood Press. Cardiovasc. Prev..

[120] Mehdipoor G., Redfors B., Chen S., Gkargkoulas F., Zhang Z., Patel M.R., Granger C.B., Ohman E.M., Maehara A., Eitel I. (2024). Hypertension, Microvascular Obstruction and Infarct Size in Patients with STEMI Undergoing PCI: Pooled Analysis from 7 Cardiac Magnetic Resonance Imaging Studies. Am. Heart J..

[121] Agita A., Thaha M. (2017). Inflammation, Immunity, and Hypertension. Acta Med. Indones..

[122] Vahldieck C., Cianflone E., Fels B., Löning S., Depelmann P., Sabatino J., Salerno N., Karsten C.M., Torella D., Weil J. (2023). Endothelial Glycocalyx and Cardiomyocyte Damage Is Prevented by Recombinant Syndecan-1 in Acute Myocardial Infarction. AJP.

[123] Civieri G., Iop L., Tona F. (2022). Antibodies against Angiotensin II Type 1 and Endothelin 1 Type A Receptors in Cardiovascular Pathologies. IJMS.

[124] Bandoni R.L., Bricher Choque P.N., Dellê H., De Moraes T.L., Porter M.H.M., Da Silva B.D., Neves G.A., Irigoyen M.-C., De Angelis K., Pavlov V.A. (2021). Cholinergic Stimulation with Pyridostigmine Modulates a Heart-Spleen Axis after Acute Myocardial Infarction in Spontaneous Hypertensive Rats. Sci. Rep..

[125] Neckář J., Alánová P., Olejníčková V., Papoušek F., Hejnová L., Šilhavý J., Behuliak M., Bencze M., Hrdlička J., Vecka M. (2021). Excess Ischemic Tachyarrhythmias Trigger Protection against Myocardial Infarction in Hypertensive Rats. Clin. Sci..

[126] Mambrini S.P., Menichetti F., Ravella S., Pellizzari M., De Amicis R., Foppiani A., Battezzati A., Bertoli S., Leone A. (2023). Ultra-Processed Food Consumption and Incidence of Obesity and Cardiometabolic Risk Factors in Adults: A Systematic Review of Prospective Studies. Nutrients.

[127] Martínez-González M.A., Gea A., Ruiz-Canela M. (2019). The Mediterranean Diet and Cardiovascular Health: A Critical Review. Circ. Res..

[128] Badimon L., Vilahur G., Padro T. (2010). Nutraceuticals and Atherosclerosis: Human Trials. Cardiovasc. Ther..

[129] Babio N., Toledo E., Estruch R., Ros E., Martínez-González M.A., Castañer O., Bulló M., DPharm D.C., Arós F., Gómez-Gracia E. (2014). Mediterranean Diets and Metabolic Syndrome Status in the PREDIMED Randomized Trial. CMAJ.

[130] Esposito K., Marfella R., Ciotola M., Giugliano F., Giugliano G., D’Armiento M., D’Andrea F., Giugliano D. (2004). Effect of a Mediterranean-Style Diet on Endothelial Dysfunction and Markers of Vascular Inflammation in the Metabolic Syndrome. JAMA.

[131] Magnoni M., Scarano P., Vergani V., Berteotti M., Gallone G., Cristell N., Maseri A., Cianflone D. (2020). Impact of Adherence to a Mediterranean Diet Pattern on Patients with First Acute Myocardial Infarction. Nutr. Metab. Cardiovasc. Dis..

[132] Zununi Vahed S., Barzegari A., Zuluaga M., Letourneur D., Pavon-Djavid G. (2018). Myocardial Infarction and Gut Microbiota: An Incidental Connection. Pharmacol. Res..

[133] Kodama S., Tanaka S., Heianza Y., Fujihara K., Horikawa C., Shimano H., Saito K., Yamada N., Ohashi Y., Sone H. (2013). Association between Physical Activity and Risk of All-Cause Mortality and Cardiovascular Disease in Patients with Diabetes. Diabetes Care.

[134] Naci H., Salcher-Konrad M., Dias S., Blum M.R., Sahoo S.A., Nunan D., Ioannidis J.P.A. (2019). How Does Exercise Treatment Compare with Antihypertensive Medications? A Network Meta-Analysis of 391 Randomised Controlled Trials Assessing Exercise and Medication Effects on Systolic Blood Pressure. Br. J. Sports Med..

[135] Ozemek C., Laddu D.R., Arena R., Lavie C.J. (2018). The Role of Diet for Prevention and Management of Hypertension. Curr. Opin. Cardiol..

[136] Chen C.-W., Chang C.-W., Lin Y.-C., Chen W.-T., Chien L.-N., Huang C.-Y. (2023). Comparison of Clinical Outcomes of Angiotensin Receptor Blockers with Angiotensin-Converting Enzyme Inhibitors in Patients with Acute Myocardial Infarction. PLoS ONE.

[137] Tucker W.J., Fegers-Wustrow I., Halle M., Haykowsky M.J., Chung E.H., Kovacic J.C. (2022). Exercise for Primary and Secondary Prevention of Cardiovascular Disease. J. Am. Coll. Cardiol..

[138] Puhl S.-L., Müller A., Wagner M., Devaux Y., Böhm M., Wagner D.R., Maack C. (2015). Exercise Attenuates Inflammation and Limits Scar Thinning after Myocardial Infarction in Mice. Am. J. Physiol. Heart Circ. Physiol..

[139] Paolisso P., Bergamaschi L., Santulli G., Gallinoro E., Cesaro A., Gragnano F., Sardu C., Mileva N., Foà A., Armillotta M. (2022). Infarct Size, Inflammatory Burden, and Admission Hyperglycemia in Diabetic Patients with Acute Myocardial Infarction Treated with SGLT2-Inhibitors: A Multicenter International Registry. Cardiovasc. Diabetol..

[140] Boden W.E., Bhatt D.L., Toth P.P., Ray K.K., Chapman M.J., Lüscher T.F. (2020). Profound Reductions in First and Total Cardiovascular Events with Icosapent Ethyl in the REDUCE-IT Trial: Why These Results Usher in a New Era in Dyslipidaemia Therapeutics. Eur. Heart J..

[141] Budoff M.J., Bhatt D.L., Kinninger A., Lakshmanan S., Muhlestein J.B., Le V.T., May H.T., Shaikh K., Shekar C., Roy S.K. (2020). Effect of Icosapent Ethyl on Progression of Coronary Atherosclerosis in Patients with Elevated Triglycerides on Statin Therapy: Final Results of the EVAPORATE Trial. Eur. Heart J..

[142] Toso A., Leoncini M., De Servi S. (2019). Statins and Myocardial Infarction: From Secondary ‘Prevention’ to Early ‘Treatment’. J. Cardiovasc. Med..

[143] Mendieta G., Ben-Aicha S., Casani L., Badimon L., Sabate M., Vilahur G. (2020). Molecular Pathways Involved in the Cardioprotective Effects of Intravenous Statin Administration during Ischemia. Basic Res. Cardiol..

[144] Hu J., Yang C., Yang G., Du H., Zhao H., Liu H. (2022). Effects of Atorvastatin Doses on Serum Level of Procalcitonin and Predictors for Major Adverse Cardiovascular Events in Patients with Acute Myocardial Infarction: A Pilot Study and Post Hoc Analysis. Coron. Artery Dis..

[145] Wang Q., Chen Z., Guo J., Peng X., Zheng Z., Chen H., Liu H., Ma Y., Zhu J. (2023). Atorvastatin-Induced Tolerogenic Dendritic Cells Improve Cardiac Remodeling by Suppressing TLR-4/NF-κB Activation after Myocardial Infarction. Inflamm. Res..

[146] Mao Y., Koga J., Tokutome M., Matoba T., Ikeda G., Nakano K., Egashira K. (2017). Nanoparticle-Mediated Delivery of Pitavastatin to Monocytes/Macrophages Inhibits Left Ventricular Remodeling after Acute Myocardial Infarction by Inhibiting Monocyte-Mediated Inflammation. Int. Heart J..

[147] Ning Y., Huang P., Chen G., Xiong Y., Gong Z., Wu C., Xu J., Jiang W., Li X., Tang R. (2023). Atorvastatin-Pretreated Mesenchymal Stem Cell-Derived Extracellular Vesicles Promote Cardiac Repair after Myocardial Infarction via Shifting Macrophage Polarization by Targeting microRNA-139-3p/Stat1 Pathway. BMC Med..

[148] Yang C., Zeng Y., Hu Z., Liang H. (2020). PCSK9 Promotes the Secretion of Pro-Inflammatory Cytokines by Macrophages to Aggravate H/Rinduced Cardiomyocyte Injury via Activating NF-kB Signalling. Gen. Physiol. Biophys..

[149] Badimon L., Luquero A., Crespo J., Peña E., Borrell-Pages M. (2021). PCSK9 and LRP5 in Macrophage Lipid Internalization and Inflammation. Cardiovasc. Res..

[150] Marfella R., Prattichizzo F., Sardu C., Paolisso P., D’Onofrio N., Scisciola L., La Grotta R., Frigé C., Ferraraccio F., Panarese I. (2023). Evidence of an Anti-Inflammatory Effect of PCSK9 Inhibitors within the Human Atherosclerotic Plaque. Atherosclerosis.

[151] Tuñón J., Bäck M., Badimón L., Bochaton-Piallat M.-L., Cariou B., Daemen M.J., Egido J., Evans P.C., Francis S.E., Ketelhuth D.F. (2018). Interplay between Hypercholesterolaemia and Inflammation in Atherosclerosis: Translating Experimental Targets into Clinical Practice. Eur. J. Prev. Cardiol..

